# Dental Law

**Published:** 1878-03

**Authors:** 


### DENTAL LAW.

The dentists of Mississippi are making an effort for the procurement of a law to regulate the practice of dentistry in that state, and to that end they now have a bill pending in the legislature. We hope they may soon realize their desires in that respect. From some experience in the same kind of work we would suggest that the object can not be obtained without well directed and persistent effort.

